# Quantitative muscle MRI and ultrasound for facioscapulohumeral muscular dystrophy: complementary imaging biomarkers

**DOI:** 10.1007/s00415-018-9037-y

**Published:** 2018-09-06

**Authors:** Karlien Mul, Corinne G. C. Horlings, Sanne C. C. Vincenten, Nicol C. Voermans, Baziel G. M. van Engelen, Nens van Alfen

**Affiliations:** Department of Neurology and Clinical Neurophysiology, Radboud University Medical Center, PO Box 9101, 6500 HB Nijmegen, The Netherlands

**Keywords:** Facioscapulohumeral muscular dystrophy, Muscle disorders, Ultrasound, MRI, Biomarkers

## Abstract

**Objective:**

To assess the overlap of and differences between quantitative muscle MRI and ultrasound in characterizing structural changes in leg muscles of facioscapulohumeral muscular dystrophy (FSHD) patients.

**Methods:**

We performed quantitative MRI and quantitative ultrasound of ten leg muscles in 27 FSHD patients and assessed images, both quantitatively and visually, for fatty infiltration, fibrosis and edema.

**Results:**

The MRI fat fraction and ultrasound echogenicity *z*-score correlated strongly (CC 0.865, *p* < 0.05) and both correlated with clinical severity (MRI CC 0.828, ultrasound CC 0.767, *p* < 0.001). Ultrasound detected changes in muscle architecture in muscles that looked normal on MRI. MRI was better in detecting late stages of fatty infiltration and was more suitable to assess muscle edema. Correlations between quantitative and semi-quantitative scores were strong for MRI (CC 0.844–0.982, *p* < 0.05), and varied for ultrasound (CC 0.427–0.809, *p* = 0.026–*p* < 0.001).

**Conclusions:**

Quantitative muscle MRI and ultrasound are both promising imaging biomarkers for differentiating between degrees of structural muscle changes. As ultrasound is more sensitive to detect subtle structural changes and MRI is more accurate in end stage muscles and detecting edema, the techniques are complementary. Hence, the choice for a particular technique should be considered in light of the trial design.

## Introduction

Muscle imaging complements the clinical assessment of muscle disorders. It reveals patterns and severity of muscle involvement that can help guide diagnosis and track disease progression [1]. Facioscapulohumeral muscular dystrophy (FSHD) is a slowly progressive inherited muscle disorder [2]. Recent insights in its pathogenic mechanism are expected to be translated into targeted therapies soon [3, 4]. Clinical trials are expected in the upcoming years, requiring (imaging) biomarkers for the assessment of muscle involvement and disease progression [5]. Especially, in early phase trials, the use of highly responsive biomarkers would enable a smaller sample size or a shorter follow-up period, and therefore a more efficient screening of potential therapies, compared to clinical outcome measures. In FSHD, muscle MRI is currently the most frequently used imaging technique in research studies. It is able to detect and quantify fatty infiltration of muscle tissue and visualize muscle edema. The degree of fatty infiltration on MRI correlates strongly to clinical measures and it is able to capture changes over time [6–8].

Muscle ultrasound may provide an alternative that is patient-friendly, safe, fast and can be performed at the bedside [9]. Various structural changes in the muscle, such as fatty infiltration, fibrosis, or edema, produce an increase in echogenicity. Especially, the presence of fibrosis is strongly correlated with an echogenicity increase [10–12]. Increased muscle ultrasound echogenicity correlated strongly with decreased muscle strength in different neuromuscular disorders [13–15]. In Duchenne muscular dystrophy, it was shown to be sensitive to disease progression [16, 17]. A disadvantage is its inability to measure deeper layers of muscle. Limited work has been done comparing quantitative ultrasound and MRI head-to-head. Measurements of muscle thickness, length and cross-sectional area were shown to yield similar results for both techniques [18–20]. In a pilot-study on five male FSHD patients, ultrasound was shown to correlate with, but also complement MRI data [21]. The promising results of this pilot, prompted us to evaluate the properties of both techniques and compare them in a larger and clinically more diverse FSHD cohort. The aim of this study is to assess the overlap and the additional value of quantitative muscle MRI and ultrasound in leg muscles. This includes assessment of structural muscle changes, correlating imaging findings to clinical measures and comparing quantitative to semi-quantitative scores.

## Materials and methods

### Patients

We included genetically confirmed FSHD patients of 18 years and older. A genetic diagnosis of FSHD1 was defined as a D4Z4 repeat array on chromosome four of ten D4Z4 repeat units or less on a permissive haplotype, and for FSHD2 as an *SMCHD1* pathogenic variant and hypomethylation of the D4Z4 repeat array on chromosome 4q35. This study was performed in conjunction with a large MRI study on FSHD patients (*n* = 140) [6]. A random subset of patients participating in the last five months of the MRI study (between June and October 2015) additionally underwent muscle ultrasound. The selection of patients was based on the availability of the ultrasound equipment. This study was conducted according to the principles of the Declaration of Helsinki (version October 2013) and in accordance with the Medical Research Involving Human Subjects Act (WMO). The study protocol was approved by the regional medical ethics committee (CMO region Arnhem–Nijmegen). All patients signed informed consent prior to their inclusion in the study.

### MRI acquisition and analysis

**Fig. 1:**
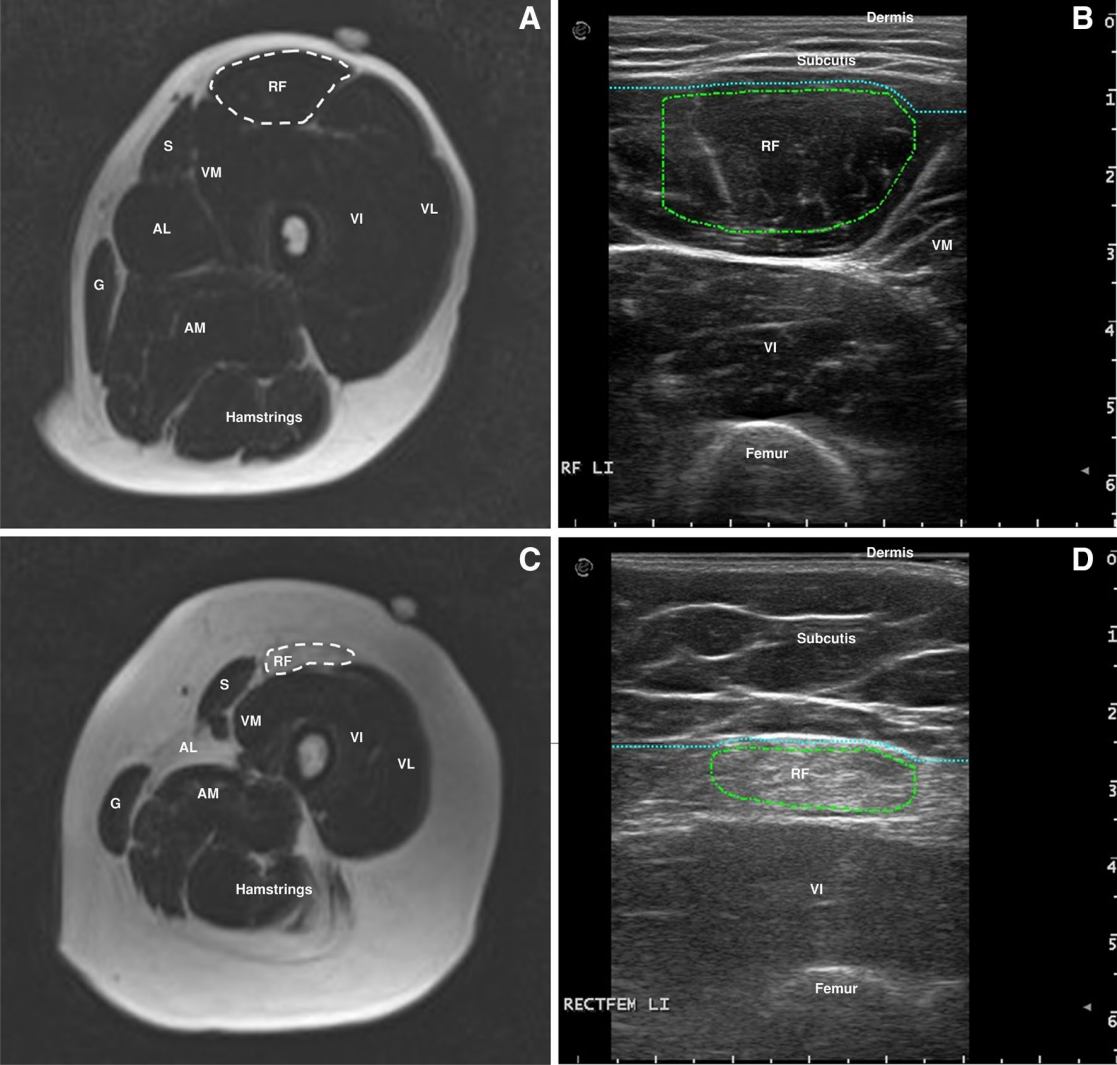
**a** MR image of left upper leg with a region of interest drawn for a normal looking rectus femoris muscle. **b** The corresponding ultrasound image to **a. c** MR image of left upper leg with a region of interest drawn for a dystrophic rectus femoris muscle. **d** The corresponding ultrasound image to **c**. S: sartorius; G: gracilis; VM: vastus medialis; VI: vastus intermedius; VL; vastus lateralis; AL: adductor longus; AM: adductor magnus

A detailed description of the MRI scanning protocol and quantitative analysis can be found elsewhere [6]. The MRI exams were performed on a 3-Tesla MR system (TIM Trio; Siemens, Erlangen, Germany). In summary, we acquired scout images in three orthogonal directions for positioning of imaging slices. Next, both legs were scanned using a Dixon 2.0 sequence and a TIRM sequence with a slice thickness of 5 mm [22]. Regions of interest (ROI) were drawn on a fat fraction map of the Dixon sequence for the muscles of interest. We made a selection of frequently affected and spared muscles in FSHD that are suited for ultrasound measurement: the rectus femoris, vastus lateralis, peroneus tertius, tibialis anterior and medial gastrocnemius bilaterally. ROI’s were drawn at specific slices using the localizer sequences: rectus femoris halfway between anterior superior iliac spine (ASIS) and upper pole of patella, vastus lateralis at two-thirds between ASIS and the upper lateral margin of patella, tibialis anterior at one-third from the inferior border of the patella to the lateral malleolus, peroneus tertius at one-fifth from the lateral malleolus to the fibular head and the medial head of gastrocnemius at one-third from the popliteal fossa to the medial malleolus. A fish oil capsule that could be visualized on the MRI images was placed on the skin to ensure that ultrasound images were obtained at the same level as the MRI images. Muscle fat fractions were calculated per ROI (Fig. 1). Fat fractions below 15% are considered normal [23]. In addition, MRI images per muscle were visually scored by one investigator (KM) using the modified Lamminen scale. Fatty infiltration was scored as: 0 = normal; 1 = mild with only traces of fatty infiltration; 2 = moderate with fatty infiltration in less than 50% of the muscle tissue; 3 = severe with fatty infiltration in more than 50% of the muscle tissue; 4 = the entire muscle replaced by abnormal signal [24, 25]. TIRM images were visually assessed for the presence of signal hyperintensity in each muscle at the location corresponding to the ultrasound images by a radiologist and one investigator (KM). The assessors of the MRI images were blinded for the ultrasound results and vice versa, but not for clinical status of the patient.

### Ultrasound acquisition and analysis

Muscle ultrasound was performed using an Esaote MyLabTwice ultrasound scanner (Esaote SpA, Genoa, Italy) using an 8–14 MHz broadband linear transducer with a 53-mm footprint, that has an axial resolution of around 0.2 mm. The ultrasound protocol was previously reported elsewhere [21]. In summary, a preset for system settings was used to ensure compatibility between measurements. Three consecutive measurements were performed to minimize variation in echo intensity. Results were averaged offline. Transverse images were acquired at the same locations as described above for the MRI. Patients were in supine position with the legs in resting position. For correct echo intensity measurements, oblique scanning angles were avoided by adjusting the angle of the probe to obtain optimal perpendicular imaging of the underlying bone. For every muscle, a ROI was drawn manually using custom software, developed at our center (Fig. 1). Raw muscle echo intensities were calculated per ROI and then converted to *z*-scores (the number of standard deviations from the mean score for sex, age and weight) using previously established reference values [26]. These *z*-scores were used for statistical analyses. *z*-scores below 2.0 (i.e., below the population 95th percentile) are considered normal.

A semi-quantitative assessment of the images was performed using the Heckmatt rating scale which ranges from one (normal echo intensity) to four (severely increased echo intensity with absent bone reflection) by an experienced neuromuscular ultrasonographer (NvA) [27]. Ultrasound images were visually inspected for textural changes of muscle tissue. We assessed ultrasound images for the presence of edema by looking for the characteristic pattern that is seen in inflammatory myopathies: a blurring of muscle architecture with “see through” echogenicity increase without decrease in echogenicity in the deeper part of the muscle [28].

### Clinical outcome measures

Disease severity was rated using the FSHD clinical score, a 15-point sum score that evaluates different muscle groups, where zero indicates no muscle weakness and 15 severe muscle weakness in all muscle groups [29]. The motor function measure (MFM) was chosen as a functional outcome measure. It assesses the severity of the motor deficit on a 32-item scale with outcomes ranging from 0 to 100% in which 100% implies no motor deficits [30]. Muscle strength was tested manually (MRC gradation [31]) for the knee extensors, foot dorsiflexors and foot plantar flexors, and quantitatively (fixed dynamometry) for the knee extensors.

### Statistical analyses

All statistical analyses were performed using IBM SPSS Statistics, version 22. Because of the skewed distribution of fat fractions and *z*-scores, a Spearman rho analysis was used to calculate bivariate correlations between MRI and ultrasound. The Spearman rho analysis was also used for correlations between quantitative and semi-quantitative scores. For correlations between clinical outcome measures and fat fractions and *z*-score, the Pearson correlation coefficient was used. To control for multiple testing, we applied the Benjamini–Hochberg false discovery rate procedure, a less conservative method than the Bonferroni correction, in which we accepted the proportion of false discoveries to be 5% [32].

## Results

### Patients

We included 27 genetically confirmed FSHD patients comprising the full spectrum of disease severity. Of these, one was an asymptomatic gene carrier who only showed minimal signs of FSHD on examination, mainly abdominal muscle weakness. This resulted in analyzing 270 lower extremity muscles. Patient characteristics are presented in Table 1. For each patient, muscle MRI and ultrasound were performed on the same day. There were no differences between FSHD1 and FSHD2 patients in either imaging or clinical outcomes.

**Table 1:** Patient characteristics

	*N* = 27
Sex	Male *n* = 17 Female *n* = 10
Age (mean ± SD [range])	53.2 years ± 12.7 [31–78]
BMI (mean ± SD [range])	26.3 kg/m^2^ ± 4.1 [21.0–35.3]
FSHD type	FSHD1 *n* = 25 FSHD2 *n* = 2
FSHD clinical score (mean ± SD [range])	5.5 ± 3.8 [14, [0–14]]
Motor function measure (mean ± SD [range])	86.6 ± 17.7 [29.1–100]

### Correlation between MRI and ultrasound

**Fig. 2:**
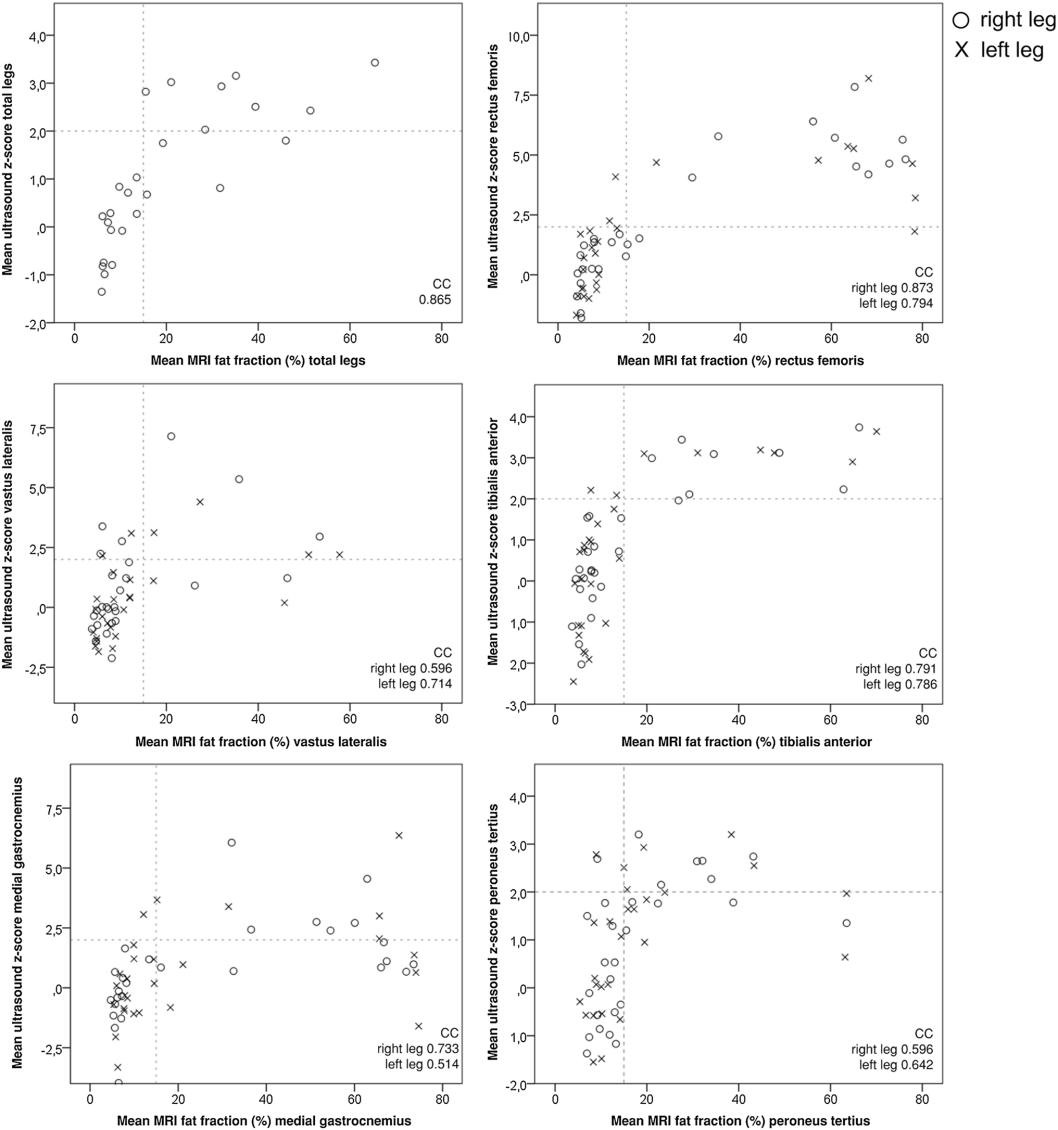
Correlation between quantitative MRI (fat fraction) and ultrasound (*z*-score) for the mean values of all muscles and for each muscle separately. The fat fractions and *z*-scores for the total legs were calculated by averaging the scores for the ten muscles measured. Dotted lines indicate limits of values that are considered normal. CC: correlation coefficient

The mean MRI fat fraction of all muscles per patient correlated highly and significantly with the mean ultrasound *z*-score with a correlation coefficient of 0.865 (*p* < 0.001). When correlating MRI fat fraction and ultrasound *z*-score per muscle for both legs separately, all correlations were statistically significant (*p* < 0.05 after correction for multiple testing), but correlation coefficients varied widely from 0.514 to 0.873 (Fig. 2). As can be noted in Fig. 2, in all muscles measured except for the tibialis anterior, the ultrasound *z*-scores often decrease towards normal at muscle sites with the highest fat fractions on MRI. In approximately 15% of all muscles, there was a high MRI fat fraction but a normal ultrasound *z*-score, or vice versa.

**Fig. 3:**
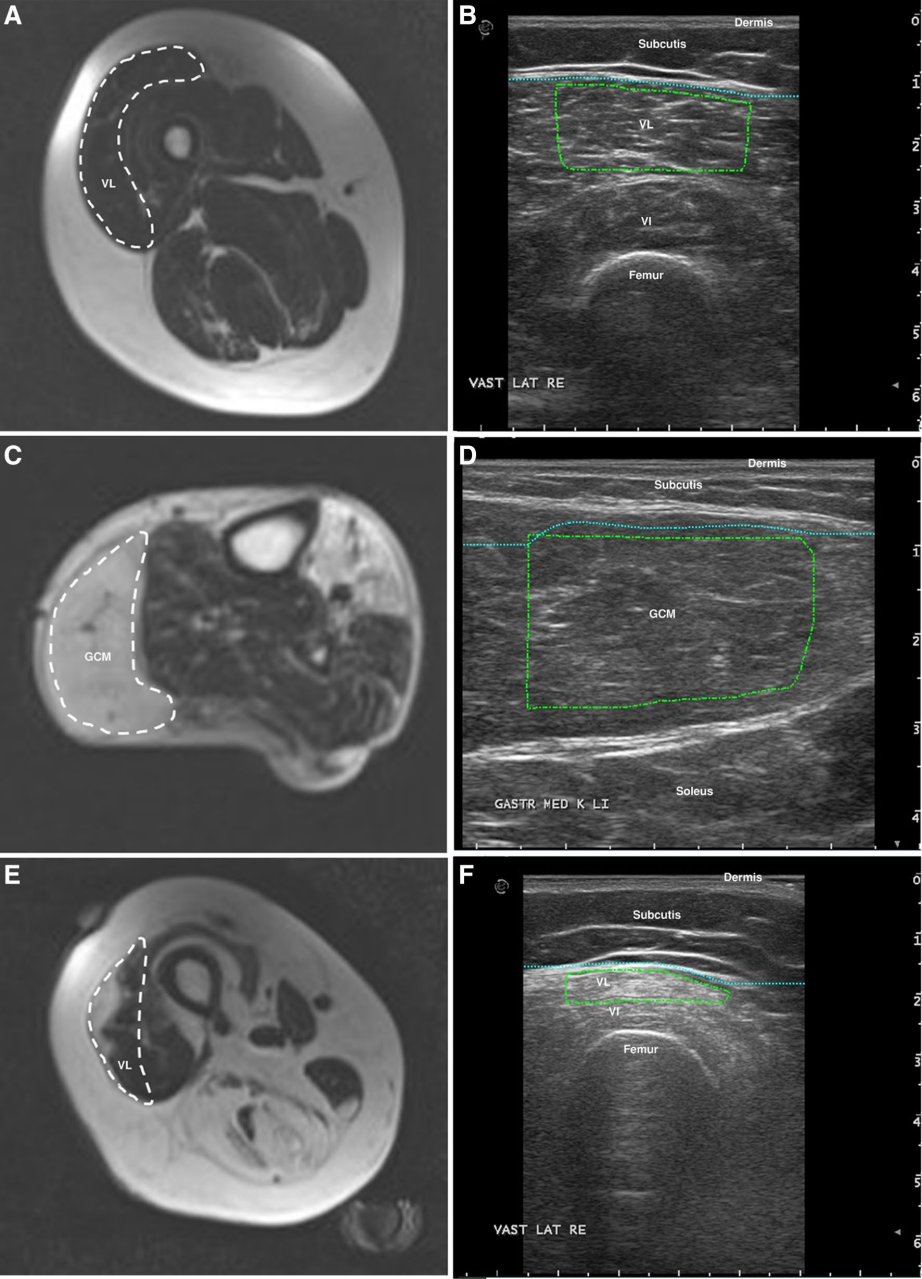
**a, b** Images of the right vastus lateralis, appearing normal on MRI (fat fraction 6%) but showing textural changes tissue texture changes (an increased amount of short linear reflective structures) on ultrasound images, resulting in a high echo intensity (*z*-score 3.4). **c, d** MRI of the left gastrocnemius medialis that is completely fatty infiltrated (fat fraction 74%) and the corresponding ultrasound image that shows a low echo intensity (resulting in a normal *z*-score of 0.6), but a disturbed architecture of the muscle tissue. **e, f** MRI of the right vastus lateralis with focal fatty infiltration (fat fraction 21%) and the corresponding ultrasound image, capturing only the fatty infiltrated part of the muscle (*z*-score 7.1)

Nineteen muscles appeared (nearly) normal on the MRI, but showed increased ultrasound *z*-scores. On visual inspection of these ultrasound images, the muscles showed tissue texture changes, consisting of an increased amount of short linear reflective structures that overall increased echogenicity. Such changes are consistent with intramuscular fibrosis, especially in the absence of fatty infiltration (Fig. 3a, b) [10, 11].

Twenty of the outliers were muscles that were severely fatty infiltrated on MRI, but had normal ultrasound *z*-scores (Fig. 3c, d). On visual inspection of these ultrasound images, the muscle tissue appeared relatively hypoechogenic, but muscle architecture was disturbed on all these images. This was most often seen in the medial gastrocnemius muscle.

Five of the outliers demonstrated heterogenous involvement of the muscle. The ultrasound measurement was performed at a site where only fatty infiltrated or only normal looking muscle tissue was present. In contrast, the MR image included the total cross-sectional area of the muscle (Fig. 3e, f).

Asymmetrical muscle involvement was found in 25/135 (18.5%) of all muscle pairs. In 14 muscle pairs, there was asymmetry both on MRI and on ultrasound images. In 11 muscle pairs there was asymmetry only on images of one of the two techniques and these were the muscles where MRI and ultrasound showed different results as described above.

### Muscle edema

The MRI showed TIRM hyperintense areas, indicating muscle edema, in 22 (8%) of 270 muscles. Two of these 22 TIRM positive muscles, both tibialis anterior muscles, also scored positively for inflammation on muscle ultrasound. Another 32 muscles were also scored positive for inflammation on muscle ultrasound, but were TIRM negative on MRI. These 32 muscles all had a fat fraction below 15% except for one medial gastrocnemius (fat fraction 18.2%). In 29 out of 32 muscles, this involved the vastus lateralis, rectus femoris or medial gastrocnemius muscles. The 19 outliers mentioned above, showing an increased *z*-score on ultrasound images, but normal fat fraction on MRI images, were all TIRM negative. TIRM hyperintense areas were found both in muscle with and without fatty infiltration.

### Correlation between imaging results and clinical outcome measures

Both the mean MRI fat fraction and mean ultrasound *z*-score of all muscles correlated strongly with the FSHD clinical score, a measure of disease severity (correlation coefficients 0.828 and 0.767, respectively, *p* < 0.001) and with the motor function measure, a functional outcome measure (correlation coefficients − 0.826 and − 0.674, respectively, *p* < 0.001). Correlations of muscle strength testing with the MRI fat fraction and ultrasound *z*-score of the corresponding muscle group are shown in Table 2.

**Table 2:** Correlations between muscle strength testing and imaging outcomes of the corresponding muscle groups

Clinical outcome	Muscle	MRI fat fraction		Ultrasound *z*-score	
		CC	*p* value	CC	*p* value
MMT dorsiflexors	Tibialis anterior				
	Right	− 0.882	0.01	− 0.772	0.01
	Left	− 0.886	0.01	− 0.729	0.01
MMT plantar flexors	Gastrocnemius medialis				
	Right	− 0.323	0.16	− 0.580	0.01
	Left	− 0.378	0.08	− 0.569	0.01
MMT knee extensors	Vastus lateralis				
	Right	− 0.823	0.01	− 0.543	0.01
	Left	− 0.828	0.01	− 0.569	0.01
	Rectus femoris				
	Right	− 0.500	0.02	− 0.335	0.14
	Left	− 0.535	0.01	− 0.230	0.30
QMT knee extensors	Vastus lateralis				
	Right	− 0.603	0.01	− 0.492	0.03
	Left	− 0.522	0.02	− 0.232	0.32
	Rectus femoris				
	Right	− 0.385	0.08	− 0.289	0.23
	Left	− 0.123	0.57	− 0.159	0.46

*p* values corrected for multiple testing

MMT: manual muscle testing; QMT: quantitative muscle testing; CC: correlation coefficient

We separately evaluated the clinical involvement of the 19 muscles showing an increased *z*-score on ultrasound images, but normal fat fraction on MRI images. Manual muscle testing of the corresponding muscle group was normal in all but two patients. One patient with increased ultrasound *z*-score of the gastrocnemius medialis muscle had mild weakness of the plantar flexors (MRC 4), but also had a fatty infiltrated soleus muscle. One patient with a high *z*-score for the vastus lateralis muscle scored MRC4 for knee extensors, but additionally showed fatty infiltration of other parts of the quadriceps muscle.

TIRM hyperintense areas were found both in muscle with normal and with decreased strength. In muscles with decreased strength, there was a combination of fatty infiltration and TIRM hyperintensity.

### Correlation between quantitative and semi-quantitative rating scales

MRI quantitative muscle fat fractions correlated moderately to strongly with the semi-quantitative modified Lamminen score for all muscles (correlation coefficient ranging from 0.641 to 0.891, *p* < 0.01 corrected for multiple testing). Correlations of quantitative ultrasound and the semi-quantitative Heckmatt score, were less strong, but still significant (range 0.418–0.840, *p* < 0.05 corrected for multiple testing) for all muscles. Moderate correlations were found for left gastrocnemius and vastus lateralis muscle. All correlation coefficients are presented in Table 3.

**Table 3:** Correlations between quantitative and semi-quantitative scores per muscle for both MRI and ultrasound images

Muscle	MRI fat fraction vs Lamminen score		Ultrasound *z*-score vs Heckmatt score	
	CC	*p* value	CC	*p* value
Rectus femoris				
Right	0.865	0.01	0.717	0.01
Left	0.818	0.01	0.763	0.01
Vastus lateralis				
Right	0.795	0.01	0.651	0.01
Left	0.762	0.01	0.418	0.01
Tibialis anterior				
Right	0.835	0.01	0.840	0.01
Left	0.835	0.01	0.749	0.01
Gastrocnemius medialis				
Right	0.891	0.01	0.677	0.01
Left	0.745	0.01	0.582	0.01
Peroneus tertius				
Right	0.652	0.01	0.742	0.01
Left	0.641	0.01	0.805	0.01

*p* values corrected for multiple testing

## Discussion

This study of 270 leg muscles of 27 patients with different stages of FSHD showed that both quantitative muscle MRI and quantitative ultrasound correlated strongly with clinical disease severity and with most of the manual muscle strength testing. There was a strong correlation between the degree of fatty infiltration on MRI and the ultrasound echo intensity. The head-to-head comparison provided unique insights into the strengths and pitfalls of both techniques. While for most muscles the techniques yielded similar results, there were differences between the two techniques in approximately 15% of the muscles measured.

For some muscles ultrasound detected changes suggestive of intramuscular fibrosis, leading to increased echogenicity, while MR images were still normal both visually and quantitatively. Whereas MRI only detects fatty infiltration and edema, ultrasound also detects fibrosis. A study on FSHD patients using muscle biopsies from tibialis anterior and vastus lateralis muscles that appeared normal on MRI, showed mild to moderate fibrosis in 11 of 17 biopsies (65%) (unpublished data). Two animal studies have shown that fibrosis leads to increased muscle echo intensity [10, 12]. However, as we did not perform muscle biopsies in this study, there is no direct pathological evidence to attribute the disturbed muscle architecture to fibrosis. Altogether, our findings suggest that muscle involvement in FSHD starts even before signs of fatty infiltration or edema become apparent on MRI, and that MRI is not able to detect all structural muscle changes. Whether these changes on ultrasound have an effect on muscle strength could not be determined, because the muscles involved perform their function as a part of a larger muscle group. Longitudinal data are necessary to determine the evolvement of these changes in muscle architecture. For clinical trials on therapeutic agents intended to slow down disease progression, patients with early disease stage are an important target group. For this particular group, quantitative ultrasound may be a more suitable biomarker than MRI.

In contrast, in muscles that were nearly completely replaced by fat on MRI, ultrasound often failed to detect the degree of abnormality. This can be explained by the fact that in severely fatty infiltrated muscles, there will be few tissue transitions left to reflect the ultrasound beam, resulting in a relatively hypoechoic image. However, on visual inspection, the ultrasound images showed an abnormal muscle texture. Thus, in these cases, new techniques for texture analysis might provide an increased detection of muscle involvement over grayscale/echogenicity analysis [33]. This difference between techniques was observed most frequently in the medial gastrocnemius muscle, that often shows early and severe involvement in FSHD [6]. For the calf muscles, the misleading decrease in echogenicity in severely affected muscles has been described earlier [34], but the current study shows that this is also the case for other muscles, such as the rectus femoris, vastus lateralis and peroneus tertius. In longitudinal studies on severely affected muscles, quantitative ultrasound measurements bare the risk of false positive results, since a decrease in *z*-score could be due to the muscle becoming more fatty infiltrated. In this particular patient group, muscle MRI would be the preferred biomarker.

A small proportion of all muscles (0.2%) showed inhomogeneous distribution of fatty infiltration within the muscle on MRI. Here, the smaller sampling area of the ultrasound images resulted in different results between the two techniques. For most muscles in FSHD the pattern of fatty infiltration is homogeneous, although it can vary along the length of a muscle [6, 35]. For other muscle disorders with a more patchy distribution such as inflammatory myopathies this poses a risk of sampling error when imaging these with ultrasound [36]. Quantitative ultrasound is currently only able to measure superficially located muscles and can often measure only a part of the muscle. Thus, in this case, MRI has the advantage of depicting all leg muscles over their entire length.

In the assessment of muscle edema, MRI and ultrasound yielded very disparate results, with a higher proportion of muscles scored positive for edema on ultrasound images. For MRI, different sequences can be applied to discriminate between signal changes due to fatty infiltration or muscle edema. With muscle ultrasound, however, a variety of changes in the composition of the muscle tissue can produce increased echogenicity. For the assessment of edema on ultrasound images, we looked for signs that are typically seen in inflammatory myopathies. As these myopathies have a different pathogenic mechanism compared to FSHD, this definition for edema may not be suited to assess FSHD muscles. Additionally, a muscle can only be positive for edema when it is mildly fatty infiltrated, because in moderate or severely fatty infiltrated muscles there is always a decrease in echogenicity in the deeper layers of the muscle due to attenuation of the ultrasound beam, and hence no “see-through”. As there currently is no better method to discern increased echogenicity due to fat and/or fibrosis from edema, MRI remains the more suitable technique to assess muscle inflammation thus far [37].

Finally, the suboptimal correlation between quantitative and semi-quantitative ultrasound scores in this study emphasizes the challenges in scoring ultrasound images visually. Even the use of a semi-quantitative rating such as the Heckmatt scale cannot capture all variables that influence the degree of abnormality. Additionally, qualitative measures are ordinal, non-linear, measures that are not suited for parametric statistical testing. For MRI, quantitative and semi-quantitative assessments correlated strongly, but both failed to detect architectural changes in some muscles that were abnormal on ultrasound images.

The main limitation of this study was the relative small sample size. Though we included the full spectrum of disease severity, the population was limited in the sense that it did not include children or severely obese individuals. Longitudinal studies will be essential to assess evolvement of MRI and ultrasound abnormalities and their relation to one another.

In conclusion, quantitative muscle MRI and ultrasound were both able to differentiate between different degrees of muscle involvement and correlated strongly to clinical severity. At the ends of the severity spectrum, the two techniques complement each other in detecting structural muscle changes. The choice for a particular technique should therefore be carefully considered in light of the target population, the clinical or research question and muscles to be measured.
